# Dynamics of the secreted frizzled related protein Sizzled and potential implications for binding to bone morphogenetic protein-1 (BMP-1)

**DOI:** 10.1038/s41598-022-18795-4

**Published:** 2022-09-01

**Authors:** Urvashi Sharma, Sandrine Vadon-Le Goff, Karl Harlos, Yuguang Zhao, Natacha Mariano, Cecile Bijakowski, Jean-Marie Bourhis, Catherine Moali, David J. S. Hulmes, Nushin Aghajari

**Affiliations:** 1 Molecular Microbiology and Structural Biochemistry, UMR 5086 CNRS-University of Lyon, 7 passage du Vercors, 69367 Lyon, France; 2 Tissue Biology and Therapeutic Engineering Laboratory, UMR 5305 CNRS-University of Lyon, 7 passage du Vercors, 69367 Lyon, France; 3 Division of Structural Biology, The Wellcome Centre for Human Genetics, University of Oxford, Oxford, OX3 7BN UK; 4 Present Address: National Institute of Biologicals, A-32, Institutional Area, Sector 62, Noida, 201309 India

**Keywords:** Biochemistry, Structural biology

## Abstract

Sizzled (Szl) is both a secreted frizzled related protein (sFRP) and a naturally occurring inhibitor of the zinc metalloproteinase bone morphogenetic protein-1 (BMP-1), a key regulator of extracellular matrix assembly and growth factor activation. Here we present a new crystal structure for Szl which differs from that previously reported by a large scale (90°) hinge rotation between its cysteine-rich and netrin-like domains. We also present results of a molecular docking analysis showing interactions likely to be involved in the inhibition of BMP-1 activity by Szl. When compared with known structures of BMP-1 in complex with small molecule inhibitors, this reveals features that may be helpful in the design of new inhibitors to prevent the excessive accumulation of extracellular matrix that is the hallmark of fibrotic diseases.

## Introduction

Bone morphogenetic protein-1 (BMP-1, EC 3.4.24.19) is a secreted multidomain Zn-metallopeptidase of the astacin M12A subfamily which plays a key role in extracellular matrix (ECM) deposition, growth factor activation and the generation of animal form^1,2^. As a member of the BMP-1/tolloid-like family (BTPs), BMP-1 controls ECM deposition by site-specific proteolysis of procollagens, proteoglycans, prolysyl oxidases and other matrix proteins; BTPs also control growth factor activity, particularly members of the TGF-β superfamily, by proteolytic cleavage of precursor forms or growth factor antagonists^1^. One such antagonist is chordin, whose cleavage by BTPs activates growth factors BMP-2, -4 and -7, thereby controlling the chordin morphogen gradient that specifies dorso-ventral patterning during embryonic development^2–4^. In most vertebrates (with the exception of mammals) this gradient is finely tuned by the presence of the protein Sizzled (Szl) which is a potent and specific inhibitor of BTPs (*K*_i_ = 1.5 nM)^5^.

Both Szl and the related protein Crescent (which also inhibits BTPs^6^) are members of the family of secreted frizzled related proteins (sFRPs) which are known to compete for binding of Wnt proteins to Frizzled protein receptors^7,8^, albeit that whether Szl itself is a Wnt antagonist remains controversial^9–12^. All sFRPs consist of an N-terminal cysteine-rich domain (CRD, also known as Fz domain) followed by a netrin-like domain (NTR), where binding to Wnts involves mainly the CRD domain^10^. In mammals, there are five sFRPs (sFRP1-5) for which sequence comparison and phylogenetic analysis reveal two subgroups^7^, sFRP1/2/5 and sFRP3/4. These sFRPs are also found in fish, amphibians, reptiles and birds, along with Szl and Crescent, which show sequence similarities closest to sFRP1/2/5. Among sFRP1-5, only sFRP2 has been reported to affect BTPs, either by inhibiting^13,14^ or enhancing^15,16^ activity, with others finding no effect at all^5,17^.

In view of their role in the excess ECM deposition that is the hallmark of fibrotic diseases, BMP-1 and other BTPs are recognized as targets for antifibrotic therapies^18^. Several small molecule hydroxamate-based inhibitors of BMP-1 have been designed and tested for efficacy in animal models, none of which have so far been approved for clinical use^19–23^. Understanding the molecular mechanism by which Szl inhibits BTP activity would therefore have implications for the development of new therapeutic strategies.

A first crystal structure of Szl from *Xenopus laevis* (PDB ID 5XGP) was recently described^9^. Here we report a new crystal structure that differs from that of Bu et al*.* by a 90° rotation of the CRD domain with respect to the NTR domain. We also present results of a molecular docking analysis of the interaction between Szl and the catalytic domain of BMP-1 (PDB ID 3EDH)^24^, following earlier studies^5^, that yield new insights into the key interactions involved.

## Methods

### Expression vectors and cloning

Native full-length Szl with a C-terminal 6xHistidine tag^5^ was expressed in pHLsec^25^ by cloning between the AgeI and KpnI restriction sites. To produce Se-Met labeled Szl, two more constructs were prepared, using the same restriction sites, with pURD^26^ and modified pHLsec (from the Oxford Protein Production Facility, OPPF) both of which include a rho-1D4 tag for enrichment of low expressing proteins. Primers used for sequencing were as follows:

- pHLsec-forward: 5′-GCTGGTTATTGTGCTGTCTCATC-3′
- pHLsec-reverse: 5′-CACCAGCCACCACCTTCTGATAG-3′
- pURD-forward: 5′-GCTGGTTGTTGTGCTGTCTCATC-3′
- pURD-reverse: 5′-GGAAGCAATAGCATGATACA-3′

### Protein expression and purification

Native Szl was produced by transient transfection of HEK 293T cells^25^. Cells (obtained from “Cellulonet—SFR Biosciences” Lyon, France) were first grown in DMEM medium supplemented with 10% FBS in 10-step cell stacks (Corning, USA) at 37 °C with 5% CO_2._ When cells were at 80% cell confluency, the Szl plasmid was introduced using the transfection agent polyethylenimine (PEI) with a DNA:PEI ratio of 1:3 (w/w). Conditioned media containing the secreted Szl protein was collected 3- and 5-days post transfection. Protease inhibitors NEM (2 mM) and Pefabloc (0.2 mM) were added to the harvested medium followed by centrifugation at 10,000*g* for 15 min at 4 °C. The clear supernatant was then dialyzed against 50 mM Na phosphate buffer pH 8.0, 0.3 M NaCl and mixed with Ni–NTA resin (pre-equilibrated in the same buffer). After 2 h of incubation at 4 °C on a rotating shaker, BioRad columns were manually loaded then washed and eluted with a ~ 200–250 mM linear imidazole gradient. Szl eluate was then dialyzed against 20 mM MES pH 6.0, 0.5 M NaCl (buffer optimised by thermal shift assay at the CTPF Platform, Paris-Saclay), concentrated using a Centricon 70 spin column (Millipore) then further purified on a Superdex 75 10/300 Increase column (GE Healthcare). Typical yields of purified Szl were 1.5 mg/L of culture medium. Purified protein was then checked by SDS-PAGE and MALS-SEC.

For Szl-SeMet, both the pURD-1D4 and pHLSec-1D4 vectors containing Szl inserts were used to generate stable and transiently transfected 293T cell lines using the robotic cell culture facility at STRUBI, Oxford, UK. Briefly, HEK cells (293 T) were grown in normal DMEM medium containing 10% FBS in roller bottles (Greiner Bio-One; 250 mL × 12). When 80% confluent, cells were transfected with pURD-Szl-1D4 and pHLSec-szl-1D4 plasmids using PEI as transfection reagent with a DNA:PEI ratio of 1:2 (0.5 mg DNA/roller bottle) then further grown in DMEM containing 2% FBS for 24–48 h in a rolling incubator. For Se-Met labelling, first the culture medium was removed then cells were gently washed with PBS. Freshly prepared labelling medium (250 mL methionine-free DMEM, 2% FBS, l-glutamine and non-essential amino acids) supplemented with 0.5 mL of l-Se-methionine (SeMet, 20 mg/mL stock, Eburon Organics) was then added to each roller bottle to give a final concentration of 40 mg/L SeMet. Cells were incubated for 4 days then conditioned media was collected. To the 6 L of the harvested medium, protease inhibitors NEM (2 mM) and PMSF (0.2 mM) were added followed by centrifugation at 3000 rpm, 4 °C for 30 min. After filtering through 0.22 µm Millipore Express PLUS disposable units, medium was then loaded on a column of activated 1D4-agarose resin (manually prepared by coupling Rho-1D4 antibody to CNBr-activated Sepharose 4B (GE 17-0430-01 2.5 mL) under gravity flow overnight at 4 °C, followed by washing with 20 mM MES pH 6.0, 0.5 M NaCl. Szl-SeMet was eluted using the peptide TETSQVAPA (Genscript), at 400 µg/mL. For elution, 3 mL of the peptide solution was added to the resin with bound Szl-SeMet then shaken at 4 °C for 2 h. Three fractions of 1.5 mL each were collected in 20 mM MES pH 6.0, 0.5 M NaCl, then Szl-SeMet eluates were pooled and loaded onto a Superdex 200 10/300 Increase column (GE Healthcare) pre-equilibrated and eluted using the same buffer. Szl-SeMet eluates from peak fractions were then pooled and concentrated using Vivaspin concentrators (Sartorius) for setting up crystallization screens. The final yield of Szl-SeMet was 450 µg from 12 L of conditioned medium.

### Small angle X-ray scattering

SAXS data were collected at B21 at the Diamond Light Source UK using samples in 20 mM MES pH 6.0, 0.5 M NaCl in the concentration range 3.5–9.5 mg/mL Data were collected at 15 °C in the q range 0.015–0.4 Å^−1^ using a Pilatus 2 M detector (3 min/180 frames per sample). After buffer subtraction, concentration-normalised intensity data were extrapolated to zero concentration using *PRIMUS*^27^ then P(r) analysis was carried out using *GNOM*^28^. Ab initio models were generated from the experimental data using *DAMMIF*^29^ then averaged and filtered using the *DAMAVER* suite^30^ to generate the final *DAMFILT* model.

### Crystallization of native Szl and Szl-SeMet

Prior to crystallization, proteins were concentrated to 12 mg/mL using Vivaspin concentrators. Initial crystals of native Szl were grown employing the sitting-drop vapour-diffusion method using 96-well plates (Greiner) at 20 °C. A Mosquito Nanolitre Robot (STP Labtech) was used to set up crystallization screens. Crystals of Szl were grown in 0.1 M NH_4_OAc and 18% PEG 3350 after incubation for 2 weeks. Diffraction quality crystals were then obtained in optimized conditions using 0.2 M NH_4_OAc, 18% PEG 3350 as precipitant. These latter were flash frozen in liquid nitrogen with 17% glycerol added to the crystallization buffer as cryo-protectant. Crystals of native Szl were reproduced in hanging drops and used for X-ray data collection.

For Szl-SeMet crystallization, screening was carried out as for Szl. Limited protein (Oxford Protein Production Facility) and PEGRx (Hampton Research, USA) screens were used for crystallization assays employing a Cartesian Technologies pipetting robot to set up 100 nL drops with a protein:precipitant ratio of 1:1. Crystals of Szl-SeMet appeared in 24 h from a crystallization buffer containing 0.1 M Bicine pH 8.5, 15% PEG 1500. Further optimizations were carried out using PEG 1500 concentrations of 17–11.3% in 0.1 M Bicine pH 8.5 in decreasing steps of 3% and with a varying protein to precipitant ratio (1:1/1:2/1:3). Large crystals of Szl-SeMet were obtained with 17% PEG 1500 which grew to full size within 3 days. The quality of these latter deteriorated within 5 days. Szl-SeMet crystals were flash-frozen in liquid nitrogen using reservoir solution supplemented with 20% glycerol as cryo-protectant.

### Data collection, data reduction, phasing and structure determination

Diffraction data for native and Szl-SeMet, were collected at 100 K on beamlines I02 and I03 of the Diamond Light Source (Oxfordshire, UK). Data for Szl-SeMet crystals were collected by inverse beam SAD in two sweeps starting at 0–45° and 180° apart. Native Szl crystals diffracted X-rays to 1.9 Å while for Szl-SeMet the best crystal diffracted X-rays to 3.3 Å resolution. Data were processed using the Xia2 pipeline^31^ at Diamond combining *XDS*^32^ and *POINTLESS* from the *CCP4* suite^33^. For phasing, four independent datasets from 4 different crystals were merged to enhance the anomalous signal. Heavy-atom coordinates of merged datasets for Szl-SeMet were located using *Autosol* from the *PHENIX* suite^34^ though with poor phasing power. Szl reflections were finally phased by combining a partial molecular replacement model obtained using the PDB coordinates of the cysteine rich domain (CRD) of mouse SFRP-3 (PDB ID 1IJX)^35^ and also the “search for model in map” module of the programme *MOLREP*^36^ employing the 1.95 Å native Szl dataset. Manual iterative rebuilding and fitting were done using *COOT*^37^ and model refinement was done with *phenix.refine*^38^ and *Refmac 5.6*^39^ using a combination of TLS and restrained refinement. Model geometry and stereochemistry was examined using *Molprobity*^40^.

### Molecular docking

Prior to docking, the protein preparation wizard *PrepWizard* (Schrödinger release 2018-3, Bioluminate, Schrödinger (suites2018-3/psp-v5.3), LLC, New York, NY, 2018) was used to optimize the crystal structures of Szl (determined in this study) and of the BMP-1 catalytic domain (PDB ID 3EDH)^24^. This included addition of hydrogen atoms and missing side-chains, filling missing loops, optimization of bond orders and hydrogen bonds and generation of ionized states. Water and other hetero-atoms were also removed. Restrained minimization of structures was carried out within an overall root mean square deviation (RMSD) of 0.3 from the initial energy minimized state. The *PRIME* structure prediction panel of the Schrödinger-Bioluminate suite was used for building the missing loop/linker (^138^DTLSKEYQYSYKE^150^) region connecting the CRD and NTR domains of Szl. Loops were further refined in *PRIME* to avoid any clashes. The crystal structure of Szl has two independent monomers (chains A and B) in the asymmetric unit, with the chain B structure being the most complete. Chain B was therefore further subjected to energy minimization and used for docking (with and without inter-domain linker loops) into the BMP-1 catalytic pocket.

Previously acquired data on key residues in Szl (Glu44, Asp92)^5^ and BMP-1 (Arg182)^24^ required for inhibition of BMP-1 activity were exploited to generate docking poses of the proteinase-inhibitor complex using the *PIPER* module of the Schrödinger-Bioluminate suite^41^, followed by refinement and energy minimization in *PRIME*. *PIPER* uses efficient Fast Fourier Transformation (FFT) in combination with pairwise interaction potentials using a docking code^42^ which greatly reduces the number of initial poses. *PIPER* also increases the number of near-native conformations in the initial selection of poses relative to other FFT-based docking programs. Alternatively, for docking analysis using more open structures of the BMP-1 catalytic domain in the region of the Cys65-Cys66 vicinal disulphide bond, previously generated from 3EDH by energy minimisation^43^, the ClusPro 2.0 server^44^ was used.

### Structural analysis and figure rendering

Docked poses were submitted to the Protein Interactions Calculator (PIC) webserver (http://pic.mbu.iisc.ernet.in/) to map contacts at the protein complex interface^45^, as well as to the PDBePISA v1.52 server. Parameters such as numbers of hydrogen bonds, hydrophobic residues, aromatic and ionic interactions were considered to examine the strength of the docked poses. Detection of hinge regions was performed using the *HingeProt* server^46^. Prior to calculating SAXS profiles from the crystal structures using *CRYSOL* 3.0^47^, the programmes *Modeller*^48^ and *AllosMod-FoXS*^49^ were used to complete missing regions including N- and C-termini and His-tags. Protein topology was determined using PDBsum^50^. All structure figures were generated using *UCSF Chimera*^51^ with the exception of Fig. 2, for which *PyMol* (Schrödinger, LLC) was used.

### Accession code

Coordinates and structure factors have been deposited in the Protein Data Bank under accession code 7EL5.

## Results

### Preliminary analysis

**Figure 1:**
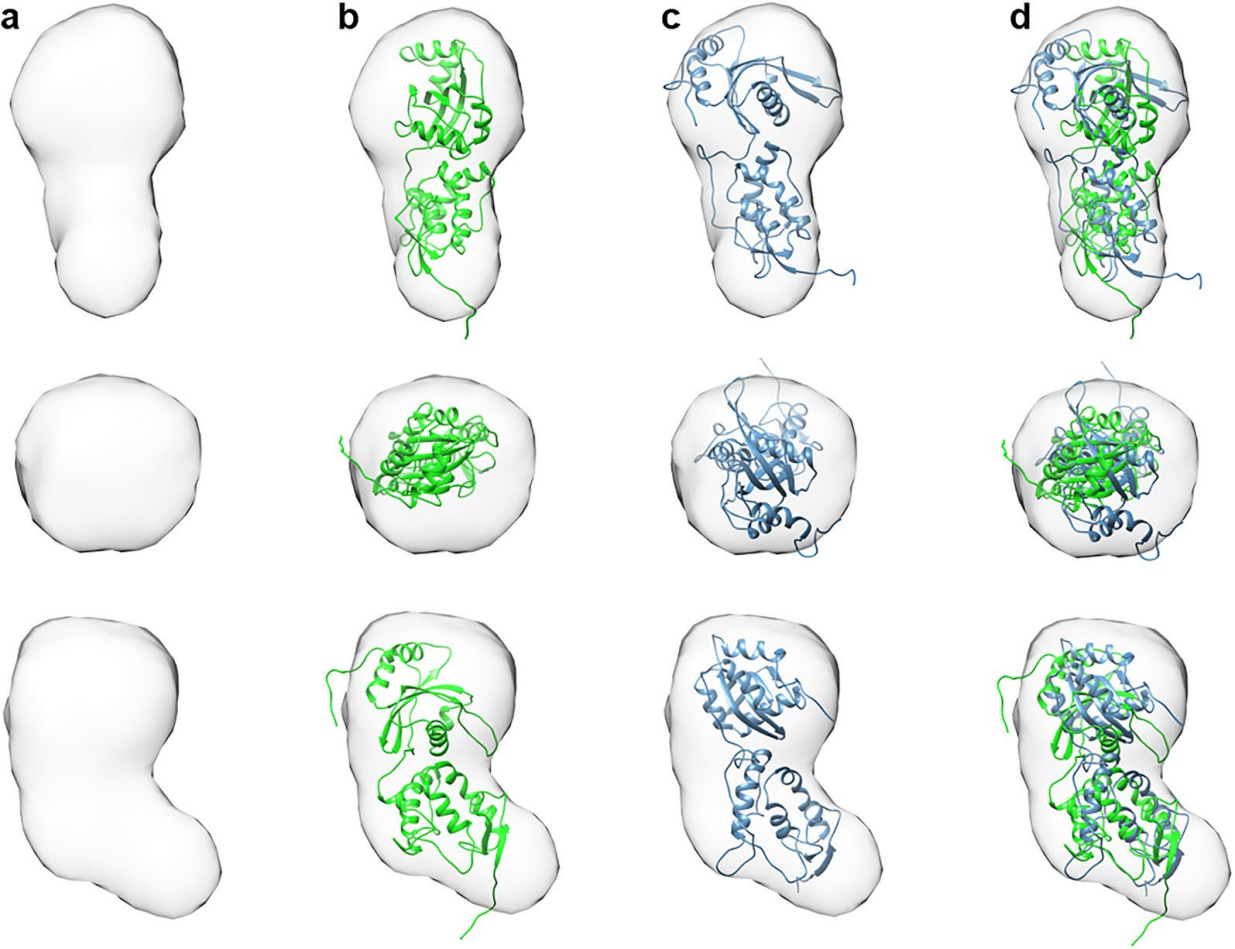
Low resolution envelope of Szl determined by SAXS with superimposed crystal structures of 5XGP and the new structure reported here (PDB-ID 7EL5). (**a**) SAXS envelope only shown in side view (top), top view (middle) and side view rotated 90° about the vertical axis (bottom). (**b**) With superimposed full length ribbon diagram of the structure reported here (in green and including modelled inter-domain linker and N- and C-terminal ends). (**c**) As B but for 5XGP (in blue). (**d**) Both structures superimposed and aligned using their N-terminal CRD domains.

By SDS-PAGE, Szl showed a single band at ~ 30 kDa in non-reducing conditions. This was confirmed by both MALS-SEC and small angle X-ray scattering, the latter showing a linear Guinier plot indicative of a monodisperse population of molecules with radius of gyration (R_g_) 24.8 Å, molecular mass 30.9 kDa and longest dimension 80 Å. Further analysis of the SAXS data revealed a bi-lobed structure consistent with the CRD and NTR domains (Fig. 1A).

### Crystal structure of Szl

Crystals from native and selenomethionine-labelled Szl belonged to space groups *P*2_1_2_1_2_1_ and *P*2_1_ and diffracted X-rays to 1.95 and 3.32 Å, respectively (Table 1). The crystal structure was determined by combining SAD phasing using the Szl-SeMet data with a partial molecular replacement model from the high-resolution native data (1.95 Å) and refinement to the same resolution. Data collection, scaling and refinement statistics for native and Szl-SeMet are summarized in Table 1. Whereas Szl was found to be a monomer in solution by both MALS-SEC and SAXS, the crystal asymmetric unit contains two molecules A and B (Supplementary Fig. S1A).

**Table 1:** Data collection and refinement statistics for native and Szl-SeMet.

	Szl-SeMet (data for phasing)	Szl (native)
PDB ID		7EL5
**Data collection**		
Wavelength (Å)	0.9796	0.9796
Resolution range (Å)	60.28–3.32 (3.41–3.32)	19.91–1.95 (2.02–1.95)
Space group	*P*2_1_	*P*2_1_2_1_2_1_
**Unit cell dimensions**		
*a*, *b*, *c* (Å)	54.99, 60.28, 75.17	63.88, 77.48, 107.49
α, β, γ (°)	90, 89.77, 90	90, 90, 90
Unique reflections	7397 (537)	74,377 (5413)
Multiplicity	21.0 (2.9)	6.8 (6.7)
Completeness (%)	99.6 (99.4)	99.0 (98.3)
Mean I/σ(I)	13.2 (1.9)	15.6 (2.3)
R_merge_	0.166 (0.471)	0.071 (1.061)
CC_1/2_		0.999 (0.825)
**Refinement**		
Protein atoms		3758
Solvent atoms		124
R_work_		0.2027
R_free_		0.2415
R.m.s.d. bonds (Å)		0.008
R.m.s.d. angles (°)		0.988
Average B-factor (Å^2^)		45.53
**Ramachandran plot**		
Favoured (%)		96.98
Allowed (%)		2.37
Outliers (%)		0.65

*R.m.s.d.* root mean square deviation.

Values in parentheses are for the highest resolution shell.

The final refined model includes two chains (A and B) containing 235 and 241 amino acids, respectively.

The new crystal structure described herein is somewhat similar to that reported earlier^9^, henceforth referred to as 5XGP, and resembles the number **8** with the N-terminal CRD domain tethered via a polypeptide linker and an interdomain S–S bridge to the C-terminal NTR domain to form an elongated shape of length 70 Å and width 30 Å (Supplementary Fig. S1B). The total buried surface area is 628 Å^2^. The structural fold of Szl is stabilized by eight disulphide bonds (seven intra-domain formed by cysteines 27–90, 37–83, 74–109, 98–136, 102–126, 159–231, 176–281 and one inter-domain formed by cysteines 115–156). In the crystal structure, the dimer interface is stabilized by a salt bridge (not present in 5XGP) between E229 (chain A) and H116 (chain B) and by an extensive set of non-bonded van der Waals interactions involving mainly Q223, L226, I227, N228, R275. No electron density was observed for the linker (residues 138–150) connecting the CRD to the NTR domain, probably due to high flexibility. Also lacking was electron density corresponding to the side-chains of residues 188–200 and 191–195 in chains A and B, respectively.

**Figure 2:**
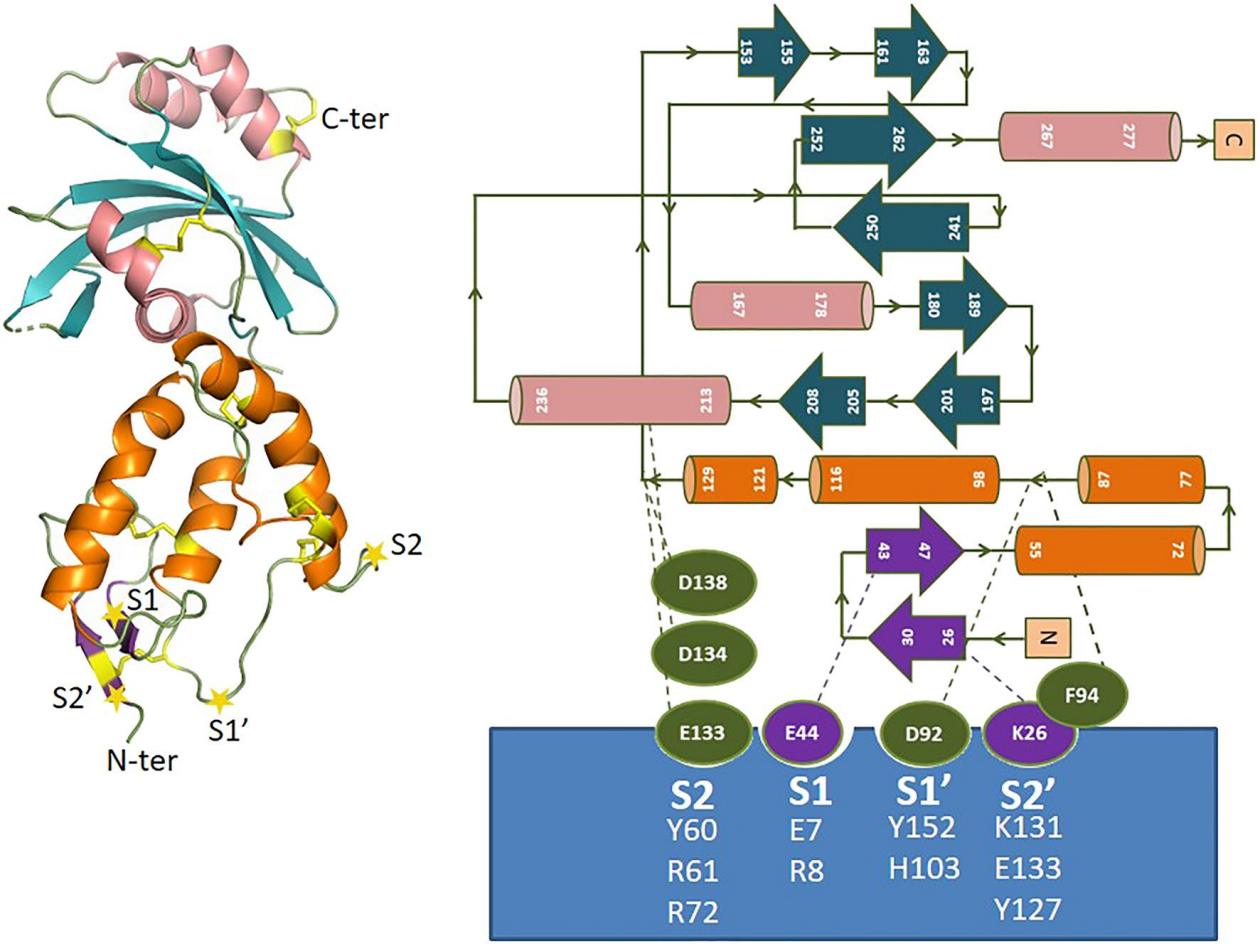
Overall fold and topology diagram of the new crystal structure of Szl. This structure (PDB-ID 7EL5), shows α-helices in orange (CRD) and salmon (NTR), β-sheets in purple (CRD) and teal (NTR) and unstructured areas and loops in green. Also shown are positions of S2′, S1′, S1 and S2 subsites in Szl referred to in the text, as well as positions of corresponding residues in Szl and interaction partners in BMP-1 (the latter in the blue box). The positions of subsites are indicated by yellow stars on the ribbon presentation.

Topological features of the Szl CRD (Fig. 2, Supplementary Figs. S1, S2) were found to be highly conserved compared to the 5XGP structure starting with two short β-strands and thereafter consisting mainly of α-helices (α1–α4). The globular fold of the CRD domain is stabilized by five disulphide bridges. In contrast, the NTR domain displays mixed β-α-β topology, consisting of seven β-strands (β3–β9), flanked by helices α5–α8. This domain is mainly stabilized by hydrophobic contacts as reported for 5XGP. The solvent accessible surface areas of the two monomers of Szl chains A and B were 12,765 and 12,588 Å^2^, respectively, perhaps due to slight differences in the orientation of each molecule. Structurally, molecules A and B aligned well with a RMSD of 1.16 Å except in the long loop at the C-terminal end of the CRD preceding the missing linker region.

**Figure 3:**
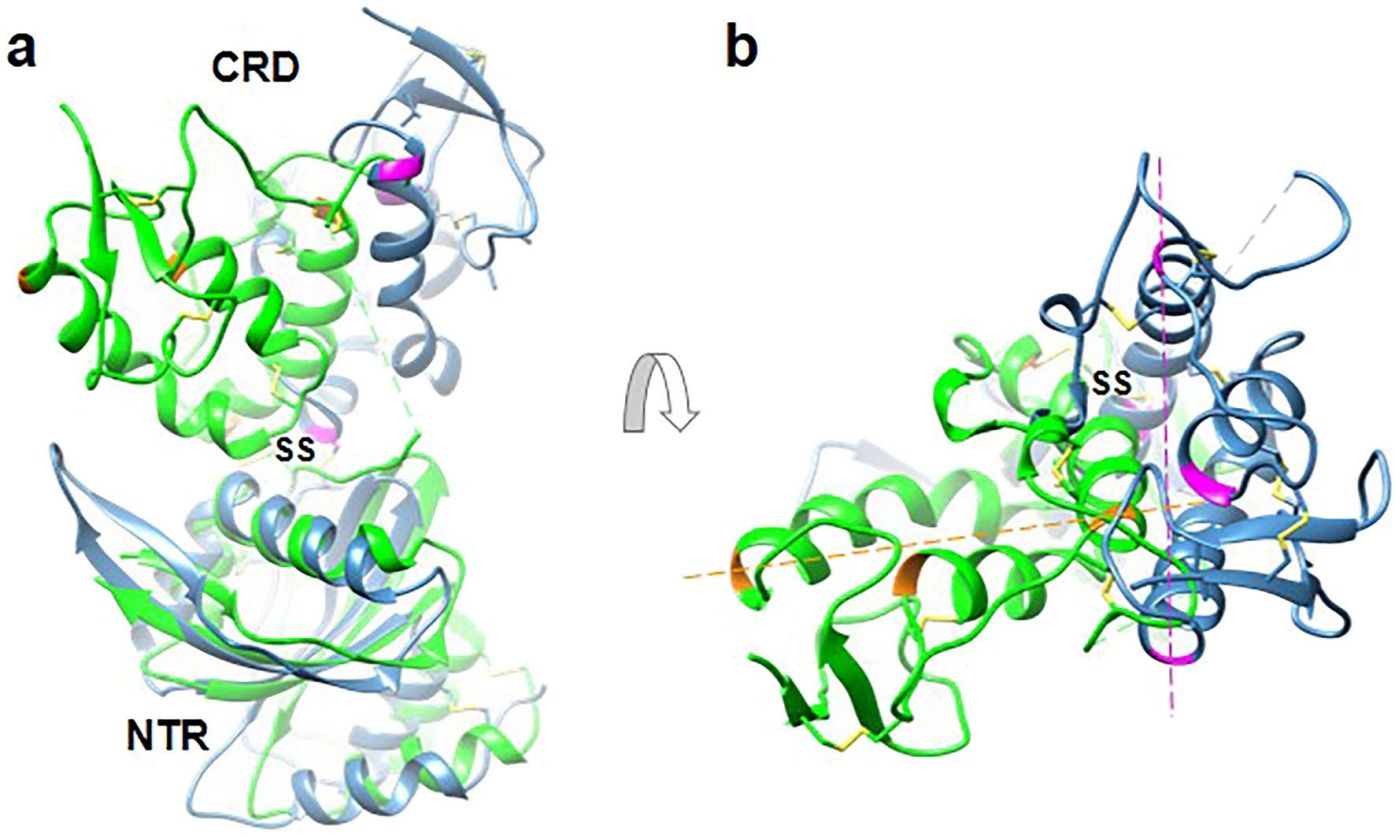
Overlay of the two crystal structures of Szl. (**a**) Side views of the new structure described here “7EL5” (green) and 5XGP (blue) aligned using their NTR domains and shown as ribbon presentations. (**b**) Top view showing the relative rotation of the CRD domains by about 90° as indicated by the dotted lines and marker residues shown in orange (new structure) and magenta (5XGP). Disulphide bridges are depicted in yellow with the inter-domain bond labelled “SS”.

Despite the above similarities when comparing individual CRD and NTR domains, striking differences were observed when the overall structure of Szl described herein was compared to 5XGP (RMSD 1.578 Å). When the NTR domains of the overall structures were aligned, there was no alignment between the corresponding CRD domains, or vice versa. Instead, there was a rotation about the long axis of one domain with respect to the other of approximately 90°, pivoting around the inter-domain disulphide bridge (Fig. 3, Supplementary Fig. S3). This was confirmed using *HingeProt*^46^ which revealed hinge residues close to and on the N-terminal side of Cys156. Even though there was a difference in the relative orientations of the CRD and NTR domains between 5XGP and the structure reported here, theoretical SAXS profiles for both structures (calculated using *CRYSOL*) were indistinguishable and consistent with the experimental SAXS data (Supplementary Fig. S4). However, difference Fourier maps (Supplementary Fig. S5) confirmed the orientation of the CRD domain with respect to the NTR domain and *vice-versa* of Szl studied herein.

### BMP-1cat/Szl complex

Several attempts were made to co-crystallize the complex between Szl and the BMP-1 catalytic domain (BMP-1cat), all of which were unsuccessful. Therefore, we made a theoretical 3D structural model of the complex by protein–protein in silico docking using the Schrödinger suite. To do so, the first step was to build the 13-residue loop comprising the linker in Szl (^138^DTLSKEYQYSYKE^150^) connecting the CRD and NTR domains (Supplementary Fig. S1B). As similar docking models/poses were obtained for the BMP-1 catalytic site both with and without the Szl CRD-NTR linker, for further analysis only the docked poses of Szl with linker were used. Analysis of the multiple poses obtained revealed that the interface between BMP-1cat and Szl was relatively stable having a free energy (ΔG) value of − 22 kcal/mol, occupying a total interactive surface area of 3626 Å^2^, and including a total of 59 hydrogen-bonds and 12 salt-bridges (Supplementary Table S1). The dynamics and stability of the theoretical BMP-1cat/Szl complex was further analyzed in a 200 ns molecular dynamics simulation run (Supplementary Methods, Supplementary Fig. S6 and Supplementary Table S2), and displayed a calculated binding free energy of ~ 10 kcal/mol being in agreement with the aforementioned calculated free energy.

Inspection of the docked poses of the BMP-1cat/Szl model complex showed that a long loop containing Szl Asp92, previously found to be essential for inhibitory activity^5,52,53^, fits neatly into the BMP-1cat active site cleft close to the catalytic zinc ion and the cysteine-rich loop containing the vicinal disulphide bond (Cys65-Cys66) (Supplementary Figs. S7, S8). This agrees with the previously established specificity of the BMP-1 S1’ pocket for substrates with Asp at the P1’ position^1,54^. This interface is stabilized by an extensive network of direct hydrogen-bonded interactions between Szl Asp92 and BMP-1 His103 and Cys66, the latter being a part of the vicinal disulphide-bridge that forms a flap at the entrance to the deep “V” shaped catalytic pocket of BMP-1. In addition, Szl Asp92 forms hydrogen bonds with BMP-1 Tyr152 and Asn128 (Supplementary Fig. S7b, Table 2) as well as charged/ionic interactions with BMP-1 His103 and His 105 at the S1′ site and with the catalytic Zn^2+^ at the active site.

**Table 2:** Interactions at the interface of the complex between the BMP-1 catalytic domain and the CRD domain of the inhibitor protein Sizzled (Szl).

BMP-1cat (3EDH)/Szl	H-bonded (up to 3.5 Å)/ionic interactions (within 5 Å)	Hydrophobic/aromatic interactions (within 5, 6 Å respectively)	Cation-π interactions (within 5 Å)
A7 (GLU)/OE2—B43 (SER)/OG	2.66		
A8 (ARG)/NE—B44 (GLU)/OE1	2.52		
A8 (ARG)/NE—B44 (GLU)/OE2	3.40		
A8 (ARG)/NH2—B44 (GLU)/OE2	2.49		
A8 (ARG)/NH2—B44 (GLU)/OE1	3.33		
*A49 (ARG)*/*NH1—B144 (TYR)/OH*	*3.11*		
*A49 (ARG)/NH1—B143 (GLU)/OE1*	*4.50*		
*A55 (TYR)/OH—B144 (TYR)/OH*	*3.24*		
A60 (TYR)/O—B134 (ASP)/OD2	3.37		
A61 (ARG)/N—B134 (ASP)/OD2	3.33		
A61 (ARG)/NE—B133 (GLU)/O	2.93		
A61 (ARG)/NH1—B136 (CYS)/N	3.21		
A61 (ARG)/NH1—B136 (CYS)/O	3.00		
A72 (ARG)/O—B41 (GLY)/O	3.38		
A72 (ARG)/NH1—B138 (ASP)/OD2	3.18		
A72 (ARG)/NH2—B138 (ASP)/OD2	2.54		
A66 (CYS)/SG—B92 (ASP)/OD2	3.18		
A103 (HIS)/ND1—B92 (ASP)/OD2	3.09		
A103 (HIS)/ND1—B92 (ASP)/OD1	3.80		
A152 (TYR)/OH—B92 (ASP)/OD2	2.66		
A128 (ASN)/OD1—B92 (ASP)/OD2	3.40		
A128 (ASN)/OD1—B93 (THR)/OG1	3.27		
A127 (TYR)/O—B25 (THR)/OG1	2.63		
A131 (LYS)/O—B25 (THR)/O	3.34		
A133(GLU)/OE1—B26 (LYS)/NZ	3.41		
A133 (GLU)/OE2—B26 (LYS)/NZ	2.52		
*A55 (TYR)—B144 (TYR)*		✓	
A62 (PRO)—B135 (MET)		✓	
A68 (TYR)—B42 (TYR)		✓	
A68 (TYR)—B89 (VAL)		✓	
A69 (VAL)—B91 (LEU)		✓	
A102 (TRP)—B91 (LEU)		✓	
A127 (TYR)—B94 (PHE)		✓	
*A49 (ARG)—B144 (TYR)*			✓
A127 (TYR)—B46 (ARG)			✓

Calculations were done using the Protein Interactions Calculator server (PIC). Interactions involving the linker region are in italics.

Apart from hydrogen-bonded and charged/ionic interactions involving catalytic site and S1′ residues, the docking also revealed significant interactions at the S2, S1, and S2′ subsites, all of which are distant from the catalytic site cleft and hence different from interaction sites normally expected for substrates (Fig. 2, Supplementary Fig. S7b and S8). The majority of these interactions involve charged/polar residues including Arg8(BMP-1)-Glu44(Szl), Glu7(BMP-1)-Ser43(Szl) at the S1 subsite near the hydrophobic pocket formed by the vicinal disulphide-bridge, while at the S2′ subsite BMP-1 Glu133 forms a salt bridge with Szl Lys26. Moreover, additional salt bridges/H-bonds at the S2 subsite involving Arg72(BMP-1) with Asp138(Szl), Arg61(BMP-1) with Glu133/Asp134(Szl) and Tyr60(BMP-1) with Glu133/Asp134 (Szl) further strengthen the positioning of Szl. The interface of the BMP-1cat-Szl theoretical complex is also stabilized by numerous face-to-face π–π interactions involving Tyr55(BMP-1)-Tyr144(Szl), Tyr127(BMP-1)-Phe94(Szl), Tyr68(BMP-1)-Tyr42(Szl) and hydrophobic contacts for Tyr68(BMP-1)-Val89(Szl), Val69(BMP-1)-Leu91(Szl), and Trp102(BMP-1)-Leu91(Szl) (Table 2, Supplementary Fig. S9). Tyr68 of BMP-1 further ensures tight binding of inhibitor residues through hydrophobic contacts at the hydrophobic surface below the S1 site (Table 2, Supplementary Fig. S7b), a feature also seen for the binding of recently reported reverse hydroxamate inhibitors to BMP-1^22^.

## Discussion

The ~ 90° rotation in the relative orientations of the Szl CRD and NTR domains, comparing the results reported here to those presented earlier^9^, was unexpected. This was particularly so in view of the inter-domain S–S bridge present in Szl, in addition to the interdomain linker, which together might be thought to constrain such movements. As described here however, there is clear evidence for hinge-bending about the inter-domain S–S bridge and linker region. To explore this further, we superimposed each crystal structure onto the sock-like SAXS low-resolution envelope. As shown in Fig. 1, both the 5XGP structure and the structure presented here fitted neatly within this shape, with the CRD domains fitting best in the thin “foot” region and the wider NTR domains in the cylindrical “leg” region. When viewed from the top (Fig. 1 middle), the relative rotations of the NTR domains are seen. Since hinge-like rotations between domains did not affect the SAXS scattering curves at the resolution of the data (~ 20 Å; Supplementary Fig. S4), all curves being additive for different shapes, it is tempting to speculate that the circular outline of the “leg” region (viewed from above) might reflect the range of conformations present in solution due to hinge movements. Such large conformational changes involving domain movements are tightly related to function in numerous proteins^55–58^. In this regard, the question of whether Szl plays a role in the inhibition of Wnt signalling remains controversial^10,11^. In the previously reported crystal structure of Szl^9^ (5XGP), it was suggested that the position of the NTR domain might prevent Wnt binding due to the tip of the extended hydrophobic palmitoleic acid (PAM) group colliding with charged residues His251 and Lys253 of the NTR domain (Supplementary Fig. S10). In contrast, with Szl 7EL5, because of the hinge rotation, the tip of the PAM group fits into the hydrophobic Leu225, Leu226 and Ile227 region of NTR, thus favouring PAM binding.

Szl residues Asp92, Phe94 and Glu44 were previously shown by site-directed mutagenesis to play key roles in the inhibition of BMP-1 activity^5^. The results presented here suggest that this list should be extended to Lys26, Glu133, Asp134 and Asp138 which are also important in anchoring Szl to BMP-1 as judged from the docking studies. Lys26 was previously suspected of being involved in binding to Szl, though its substitution by Ala was found to have little effect on inhibitory activity^5^. This underlines the prominent role played by cooperative interactions in stabilizing the complex. In addition, the flexible nature of particularly Asp92 (interaction with site S1′ in BMP-1) and E133 (interaction with site S2) in conjunction with residues interacting with sites S1 and S2′ located in secondary structure elements that are more or less superposable between the two Szl structures, points to a recognition/binding mode requiring alternating rigid anchoring points and flexible regions. Also, when superimposing the *Danio rerio* Sizzled 3D structure model as generated by Alpha-Fold2^59^ (hereafter “AF2”, publicly available on www.uniprot.org), the rotation of the NTR domain relative to the CRD domain is similar to what we have observed for 7EL5 (Supplementary Fig. S3), with residues corresponding to Asp92 and Glu133 also showing a certain flexibility. We note that *D. rerio* Szl displays 52% strict sequence identity with *X. laevis* Szl and also inhibits BTPs^60^. While Szl binds to BMP-1 via a loop tipped by Asp92 that penetrates into the active site cleft (Supplementary Figs. S7 and S8), with its tip aligned in the direction of an authentic substrate^61^, Szl itself is not cleaved by BMP-1. This is despite the fact that Asp92 is by far the most commonly found P1′ residue bound to the S1′ site for all known BMP-1 substrates^62^. Interestingly, an Asp-tipped loop also plays a key role in the inhibition of another astacin family metalloproteinase, meprin β, by the endogenous protein fetuin-B^63^. Also, when superimposing the full-length three-dimensional structure of proBMP-1 as generated by Alpha-Fold2^59^ onto the complex reported herein, it can be noted that whereas most of the BMP-1 propeptide is unstructured, the stretch containing the so-called “aspartate switch” is structured and buried in the BMP-1 catalytic domain (Supplementary Fig. S11). Intriguingly, this switch which is common to all astacin metalloproteinases co-localizes with the Asp-92 loop in Szl from our model complex, with the Asp residue at the tips in both model structures. Moreover, both loops are rich in hydrophobic residues including Phe52 from the proBMP-1 AF2 model which is located close to the critical Phe94 in Szl. Finally, we note that Asp92 in Szl is too far away (> 9 Å) to interact with Arg182 in BMP-1, formerly called Arg175 and thought to be key for binding to substrates with Asp in the P1′ position^24^. This may partially account for Szl not being cleaved by BMP-1, in addition to it binding to BMP-1 mostly by straddling the catalytic site cleft.

A comparison of the docked poses of the modelled BMP-1cat/Szl complex was also carried out with docking performed using Szl and an open conformation of BMP-1 generated by energy minimization^43^. Overall fitting and positioning of the Szl inhibitory loop near the BMP-1 catalytic site appeared to be preserved as reflected in the RMSD of 0.9 Å for aligned C-α’s. However, subtle changes/shifts were observed for residues involved in interactions close to the catalytic site (Supplementary Fig. S12a). A total shift of 2 Å was seen for the Asp92 side-chains of Szl which appears to be hydrogen-bonded to Tyr152 of BMP-1 close to the catalytic site (Supplementary Fig. S7b). This was not surprising in that Asp92 is located in a flexible loop (interacting with site S1′ in BMP-1) as observed when comparing the Szl structure determined here with that of 5XGP^9^ and in which a distance of 5.5 Å between the Cα atoms is found (Supplementary Fig. S3). Similar repositioning was seen in the cysteine rich loop containing the vicinal disulphide-bridge. To accommodate the interaction with Szl residues Lys26, Glu44 and Glu133, subtle repositioning of S2′, S1 and S2 BMP-1 subsite residues Glu133, Arg8 and Arg61, respectively, was observed indicating a flexible binding mode in the presence of the Szl inhibitor. Again, the flexible binding mode was in agreement with observations seen near the S2 site in BMP-1 when comparing our Szl structure (7EL5) with that of 5XGP; these revealed a shift of 9.2 Å between the Cα atoms of E133 in the respective structures (Supplementary Fig. S3). Concerning residues interacting with BMP-1 residues in sites S1 and S2′, as expected these are less flexible being located within more structured parts of Szl.

The catalytic subsites of BMP-1 and other Zn-dependent endopeptidases are now well established based on the crystal structures and modelling of several protease/peptide-inhibitor complexes^22,64,65^. Here we show that key residues in Szl occupy one of these subsites (S1′) as well as other subsites S2′, S1, S2 that span the catalytic site cleft (Fig. 1, Supplementary Figs. S7 and S8). In this regard, it has recently been shown^22^ that the binding to BMP-1 of small molecule hydroxamate (PDB IDs 6BTP, 6BTQ) and reverse hydroxamate (PDB IDs 6BSL, 6BSM) inhibitors can be associated with important movements in key residues of BMP-1, particularly its vicinal disulphide-bridge (Cys65–Cys66), as demonstrated most clearly with reverse hydroxamate compound 22 (PDB ID 6BSM, Supplementary Fig. S12b). These observations confirm the previously suspected flexibility of this region^24,43^. Regarding the role of the vicinal disulphide bridge in the BMP-1cat/Szl complex, Supplementary Fig. S12a shows that movements in this region similar to those observed with small molecule inhibitors may lead to changes in the relative orientations of BMP-1cat and Szl, particularly near subsite S2. Also, Szl and compound 22 (EVV) in the 6BSM crystal structure share common binding sites to BMP-1, such that conformational changes in BMP-1 residues Arg61 and Arg72 allow them to interact with both ligands (Supplementary Fig. S12b). Such conformational repositioning of subsite residues has previously been reported for other Zn-dependent endopeptidases^66^. This conformational flexibility may also contribute to the potencies of different BMP-1 inhibitors, as reflected in their *K*_i_ values of 6.8 pM for reverse hydroxamate compound 22^22^ and 1.5 nM for Szl^5^, both of which are lower than for hydroxamate-based inhibitors^19^.

In conclusion, this study reveals new structural insights into the flexibility between the two globular domains, CRD and NTR, of Sizzled. Despite its interdomain disulphide bridge, not present in other SFRPs except Crescent, Szl has remarkable freedom of movement about its CRD and NTR domains. Our studies have moreover shed light on the possible mechanism of inhibition of BMP-1 activity by the secreted frizzled related protein Sizzled. Interestingly, its likely mechanism of binding to BMP-1 has features in common with recently developed reverse hydroxamate inhibitors. These results may contribute to the development of future treatments to prevent the excess accumulation of extracellular matrix that is the hallmark of fibrotic diseases.

## Supplementary Information

Supplementary Information.

## Data Availability

Coordinates and structure factors have been deposited in the Protein Data Bank under accession code 7EL5. Other data are available from the corresponding author upon reasonable request.
