# Supplementary material for: Dynamics of the secreted frizzled related protein Sizzled and potential implications for binding to bone morphogenetic protein-1 (BMP-1)
Source: Sci Rep. 2022 Sep 1;12:14850. doi: 10.1038/s41598-022-18795-4 (PMC9437010; doi:10.1038/s41598-022-18795-4)
Supplement: Supplementary file 1 — Supplementary Information. [file 41598_2022_18795_MOESM1_ESM.pdf]

Supplementary Information

Dynamics of the secreted frizzled related protein Sizzled and potential implications for binding to bone morphogenetic protein-1 (BMP-1)

Urvashi Sharma, Sandrine Vadon-Le Goff, Karl Harlos, Yuguang Zhao, Natacha Mariano, Cecile Bijakowski, Jean-Marie Bourhis, Catherine Moali, David J.S. Hulmes and Nushin Aghajari*

Supplementary Methods

MD simulation using GROMACS

To access the stability of the BMP-1cat/Szl complex, molecular dynamics (MD) calculations were carried out. In the first step the topology of the protein-protein complex was generated by CHARMM General Forcefield (1). The second step involves solvation of the protein-inhibitor complex as well as determining the shape and size of the system. Na^+ and Cl^- ions were added in this step to neutralize the system. The complex was initially solvated in a cubic box of TIP3P waters and then Na^+ and Cl^- ions were added to neutralize the net atomic charge of the whole system by random replacement of water molecules. The NaCl concentration was set to the physiological value of 0.15 M (2). Non-bonded interactions were treated with a 12 Å cut-off distance and the neighbour searching list were buffered with the Verlet cutoff-scheme and the long-range electrostatic interactions were treated with the particle mesh Ewald (PME) method (3-4). CHARMM36m forcefield was applied on the protein-protein complex. Prior to production simulation, energy minimization of the system was carried out by using steepest descent algorithm (5000 steps). The complex was then equilibrated for stabilizing its temperature and pressure by subjecting it to NVT (constant number, volume and temperature) and NPT (constant number, pressure and temperature) ensemble and simulating for 125 ps at a temperature of 303.15 K. Finally, the complex was subjected to production simulation run for 200 ns in NPT ensemble at 303.15 K and 1 bar. To maintain the temperature and the pressure, a Nose-Hoover thermostat and a Parrinello-Rahman barostat was used, respectively. GROMACS 2020.4 was used for equilibration and production runs during all MD calculations (5). The LINCS algorithm was used for constraining H-bonds using the inputs provided by CHARMM-GUI. The V-rescale thermostat at 300 K with a coupling constant of 1 ps was employed. Molecular geometries were relaxed with the steepest descent algorithm with 50000000 steps. The trajectories were stored every 2 ps. Simulations of 200 ns in NPT assembly were performed for the MD production stage. All the molecular dynamic calculations were performed using NMRbox (https://nmrbox.org/) server platform resources (6).

Trajectory analysis

The trajectories from molecular docking simulation from the MD production stage were employed for multiple parameter analysis using the VMD molecular graphics program (7). The root mean square deviation (RMSD) of atomic positions for BMP-1cat and Szl was calculated

by fitting protein α-carbon atoms with the gmx_rms subprogram. The sub program, gmx_gyrate was used for estimating the radius of gyration of all protein atoms in the complex and number of hydrogen bonds were calculated (inside the protein-protein interface) with the gmx_hbond. Trajectories from MD production runs were converted to PBD files with an interval of 10 ns interval using gmx_trjconv for further analysis of kinetic parameters.

Binding free energy calculations

Calculation of the binding free energy (BFE) and dissociation constant (K_D) of the BMP-1cat/Szl complex was done using the online Prodigy server (https://wenmr.science.uu.nl/prodigy/) (8-9) by submitting the PDB files generated from the MD simulation trajectories of the complex at 0, 10, 20, 30, 40, 50, 60, 70, 80, 90, 100, 120, 130, 140, 150, 160, 170, 180, 190, 200 ns of the simulation run.

1. Brooks BR., *et al*. CHARMM: the biomolecular simulation program. *J. Comput Chem.* **30**;30(10):1545-614 (2009).
2. Hammad, S., Bouaziz-Terrachet, S., Meghnem, R., & Meziane, D. Pharmacophore development, drug-likeness analysis, molecular docking, and molecular dynamics simulations for identification of new CK2 inhibitors. *J. of Molecular Modeling*, **26**(6), 160 (2020).
3. Darden, T., York, D., & Pedersen, L. Particle mesh Ewald: An N·log(N) method for Ewald sums in large systems. *The J. of Chem. Phys.*, **98**(12), 10089 (1993).
4. Essmann, U., *et al*. A smooth particle mesh Ewald method. The *J. of Chem. Phys*., **103**(19), 8577 (1995).
5. Abraham, M. J., *et al*. GROMACS: High performance molecular simulations through multi-level parallelism from laptops to supercomputers. *SoftwareX*, **1–2**, 19–25 (2015).
6. Maciejewski, M.W., *et al*. NMRbox: A Resource for Biomolecular NMR Computation. *Biophys J.*, 112: 1529-1534 (2017).
7. Humphrey, W., Dalke A. & Schulten K. VMD: visual molecular dynamics. *J Mol Graph*. **14**(1):33-8, 27-8 (1996).
8. Vangone, A. & Bonvin, A.M.J.J. "Contact-based prediction of binding affinity in protein-protein complexes", *eLife*, **4**, e07454 (2015).
9. Xue, L., Rodrigues, J., Kastritis, P., Bonvin, A.M.J.J. & Vangone, A., "PRODIGY: a web-server for predicting the binding affinity in protein-protein complexes", *Bioinformatics*, doi:10.1093/bioinformatics/btw514 (2016).

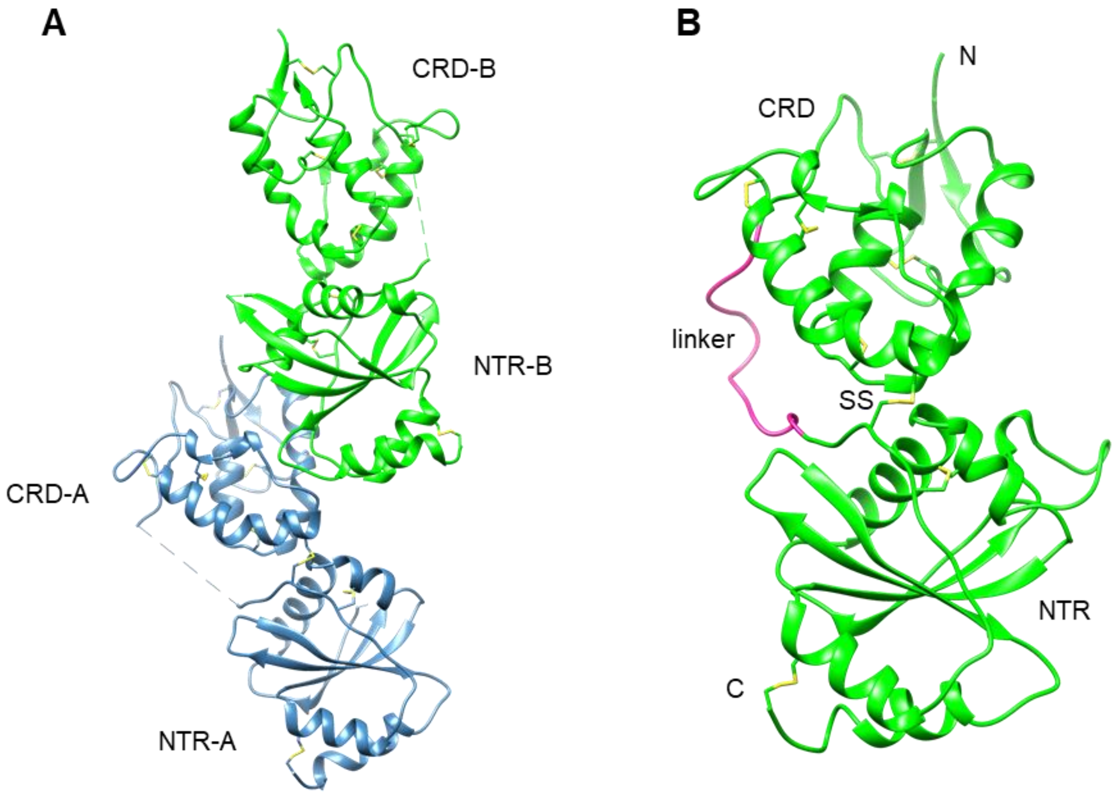

Supplementary Figure S1. Overall structure of Szl. (A). Ribbon presentations of the two molecules A and B of Szl in the crystal asymmetric unit coloured in blue and green, respectively. Missing 13-residue linkers (138-150) connecting CRD and NTR domains are indicated by dashed lines. (B) Szl chain B rendered with the linker connecting the CRD and NTR domains. The 13-residue linker (138-150) built using Schrödinger PRIME is shown in deep pink. Disulphide bridges are depicted as yellow sticks with the inter-domain bond (115-156) labelled SS. Note that the inter-domain S-S bridge was wrongly attributed to residues 115-159 in an earlier publication (Bijakowski et al., 2012), based on mass spectrometry data.

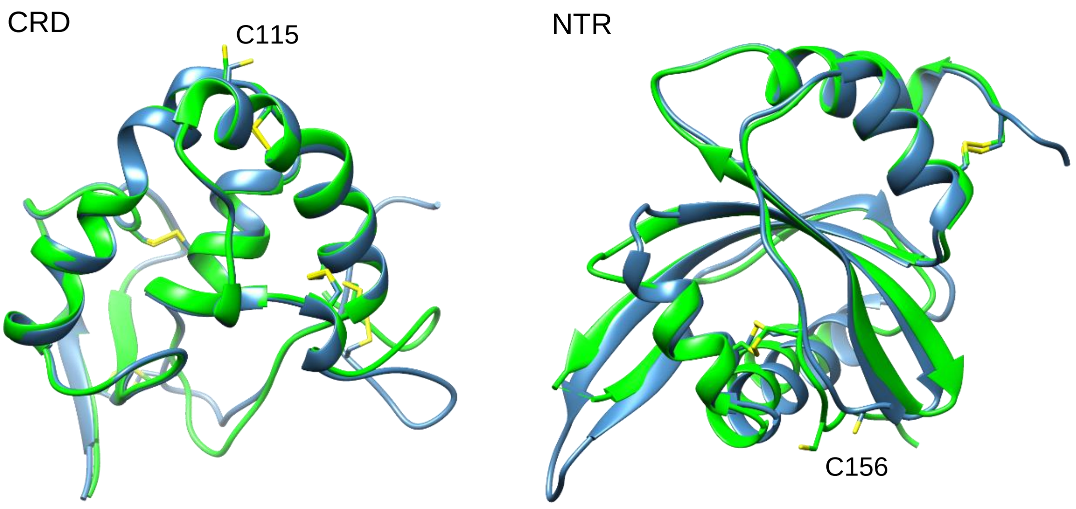

Supplementary Figure S2. Alignments of the CRD and NTR domains of the structure described here (7EL5, green) with the corresponding domains in 5XGP (blue). Intra-domain disulphide bonds are shown, as well as Cys115 and Cys156 involved in the inter-domain disulphide bond.

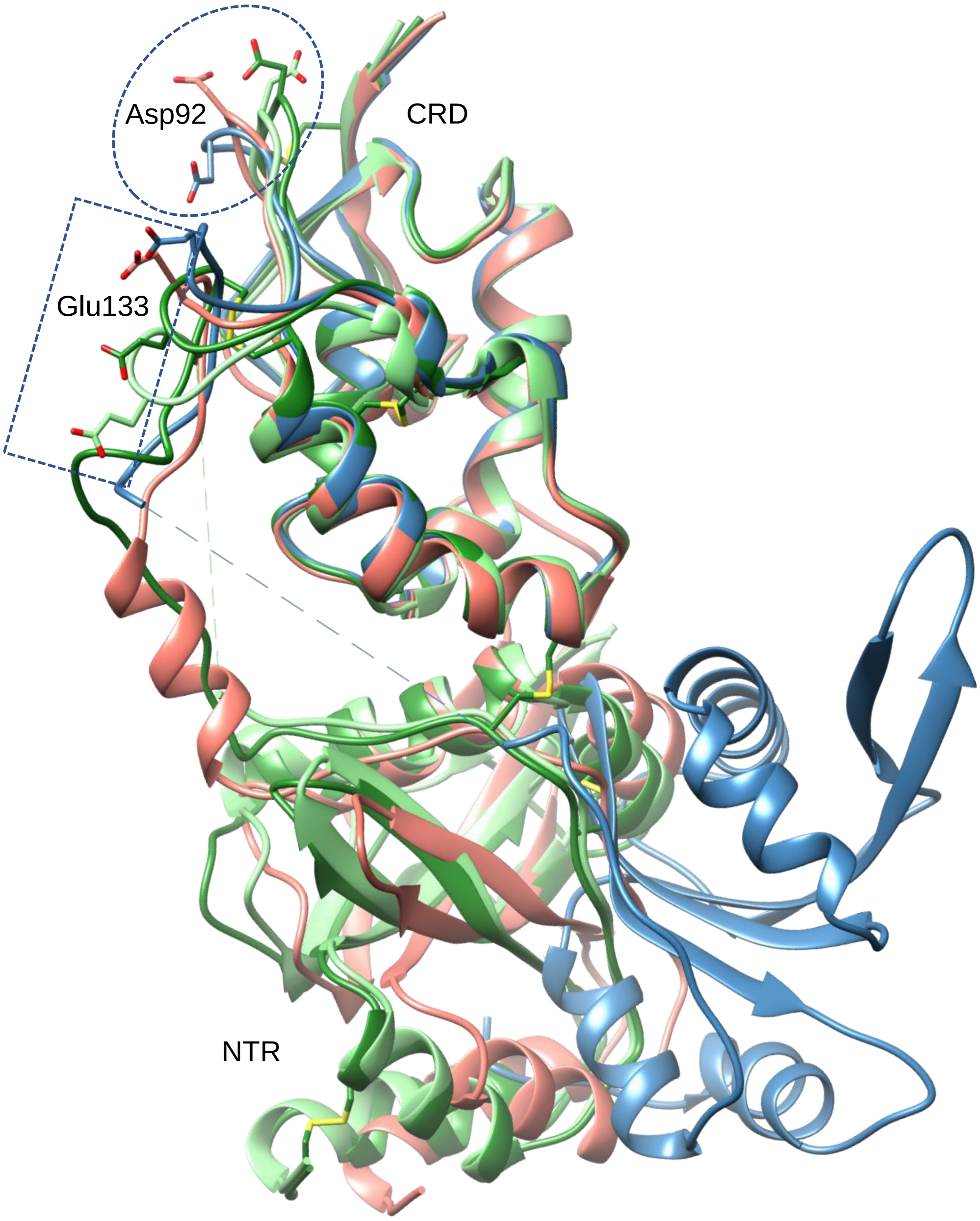

Supplementary Figure S3. Superposition of 3D structures of Szl (aligned using their CRD domains) from X-ray crystallography (7EL5 studied herein in pale green, 5XGP in blue), as part of the Szl (dark green) / BMP-1 (not shown) complex and *Danio rerio* Szl when modelled using Alpha-Fold2 (salmon colour). Also shown are the S-S bonds in 7EL5 as well as residues Asp92 and Glu133 in all structures highlighting the flexibility of the loops in this part of the CRD domain.

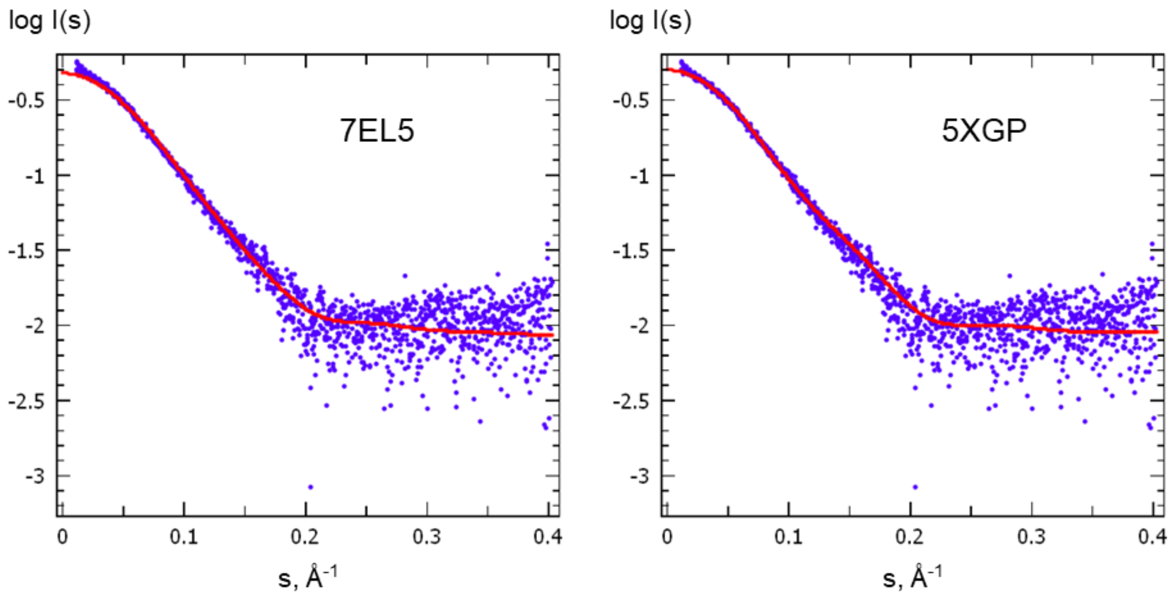

Supplementary Figure S4. Calculated small angle scattering curves (in red) based on the crystal structures of 7EL5 (data presented here) and 5XGP (Bu et al, 2017), calculated using *CRYSOL*, compared with the observed SAXS data for Szl (in blue) extrapolated to zero concentration. Despite the differences in relative orientations of the CRD and NTR domains in the crystal structures, these have little effect on the calculated small angle scattering data.

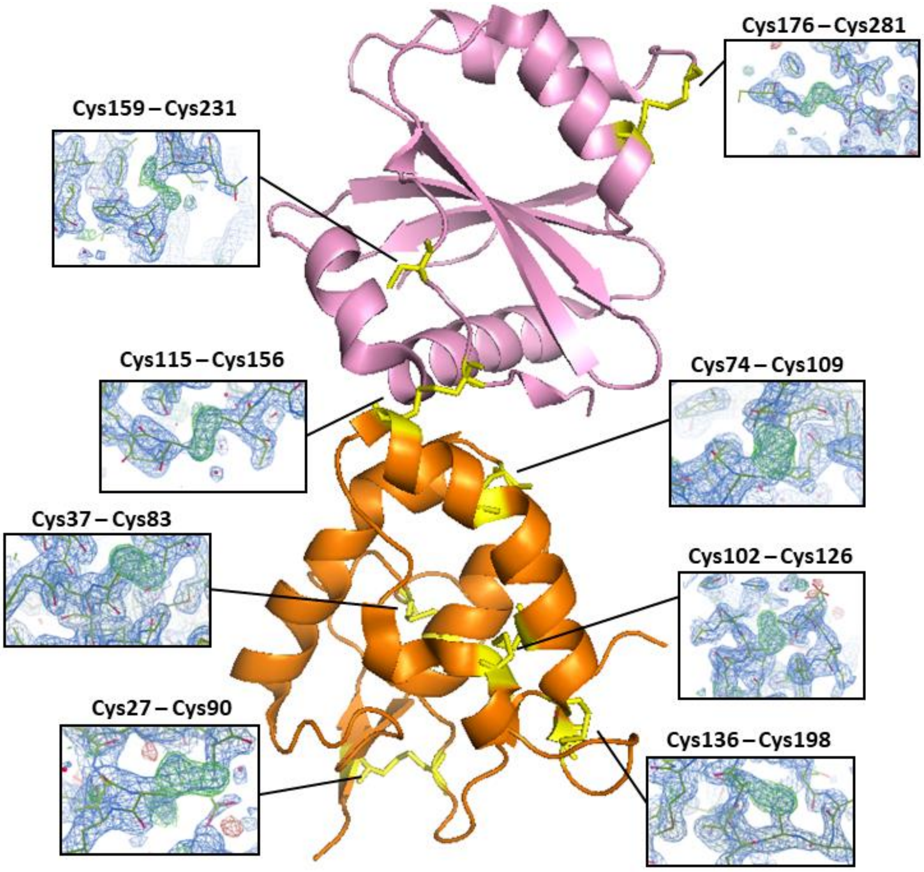

Supplementary Figure S5. Overall view of Szl (7EL5 studied herein) with the NTR domain in pink, the CRD domain in orange and S-S bridges highlighted in yellow. When these latter cysteine residues forming disulphide bridges have been substituted by alanine residues, very clear difference Fourier Fo – Fc density appears as shown in the black boxes. Herein close-up views of electron density maps with 2Fo – Fc in blue and difference Fourier Fo – Fc maps in green around the substituted alanines unambiguously indicate the presence of disulphide bridges validating the orientation of the two domains with respect to each other as seen in 7EL5.

Hydrogen bonds XML

##	Structure 1	Dist. [Å]	Structure 2
1	B:VAL 105[N]	3.31	A:GLU 7[OE1]
2	B:ASN 68[N]	2.99	A:GLY 13[O]
3	B:GLU 65[N]	3.51	A:THR 45[OG1]
4	B:CYS 231[SG]	3.26	A:GLY 64[O]
5	B:CYS 159[N]	3.57	A:CYS 65[O]
6	B:GLY 158[N]	3.04	A:CYS 66[O]
7	B:CYS 156[SG]	3.41	A:ALA 78[O]
8	B:CYS 115[SG]	2.51	A:ALA 78[O]
9	B:ALA 232[N]	3.27	A:SER 80[O]
10	B:LEU 235[N]	3.17	A:GLY 82[O]
11	B:GLN 233[N]	3.46	A:CYS 85[O]
12	B:ILE 236[N]	3.86	A:ASP 86[OD1]
13	B:LEU 226[N]	3.29	A:VAL 91[O]
14	B:GLN 223[N]	3.71	A:VAL 92[O]
15	B:GLY 252[N]	3.31	A:THR 106[O]
16	B:HIS 251[ND1]	2.28	A:ARG 107[O]
17	B:CYS 281[SG]	3.71	A:VAL 117[O]
18	B:ASP 177[N]	3.89	A:ARG 118[O]
19	B:ALA 174[N]	3.75	A:ASN 120[O]
20	B:LYS 280[N]	3.49	A:ASN 120[OD1]
21	B:ILE 249[N]	2.76	A:ASN 128[O]
22	B:SER 147[OG]	2.20	A:GLU 138[OE1]
23	B:GLU 222[N]	2.37	A:MET 150[SD]
24	B:THR 170[N]	2.74	A:ARG 159[O]
25	B:PRO 203[N]	2.72	A:GLN 181[OE1]
26	B:ALA 192[N]	3.63	A:THR 183[O]
27	B:THR 72[OG1]	3.17	A:TYR 197[OH]
28	B:CYS 74[SG]	3.49	A:TYR 197[OH]
29	B:MET 36[O]	3.46	A:CYS 43[SG]
30	B:ASP 39[O]	3.43	A:ALA 201[N]
31	B:LEU 69[O]	3.63	A:TRP 10[N]
32	B:GLN 71[OE1]	2.46	A:VAL 44[N]
33	B:GLN 71[OE1]	3.13	A:CYS 43[N]
34	B:CYS 74[SG]	2.74	A:TRP 10[N]
35	B:VAL 105[O]	3.83	A:VAL 9[N]
36	B:ALA 110[O]	3.68	A:SER 54[OG]
37	B:LEU 113[O]	2.49	A:TYR 55[N]
38	B:CYS 115[SG]	2.70	A:TYR 68[N]
39	B:GLU 150[OE2]	2.65	A:PRO 134A[N]
40	B:GLN 157[O]	3.32	A:SER 67[N]
41	B:GLY 158[O]	2.35	A:CYS 66[N]
42	B:ILE 162[O]	2.21	A:ASN 84[ND2]
43	B:LYS 169[O]	3.59	A:GLY 160[N]
44	B:LEU 172[O]	3.06	A:ILE 121[N]
45	B:GLU 173[O]	3.39	A:PRO 123[N]
46	B:ASP 177[O]	2.97	A:ARG 118[NH1]
47	B:GLN 209[O]	3.13	A:TYR 144[OH]
48	B:TYR 215[O]	3.50	A:ASP 189[N]
49	B:TYR 215[OH]	3.02	A:GLN 192[N]
50	B:ASP 216[OD1]	3.23	A:GLN 192[NE2]
51	B:GLU 222[OE1]	3.69	A:SER 186[N]
52	B:GLN 223[O]	2.64	A:VAL 98[N]
53	B:ASN 228[OD1]	3.07	A:TYR 68[N]
54	B:ILE 236[O]	2.97	A:PHE 88[N]
55	B:ILE 245[O]	3.12	A:ALA 153[N]
56	B:ASP 248[OD1]	3.37	A:LYS 131[N]
57	B:TRP 276[O]	2.60	A:ASN 120[ND2]
58	B:LYS 280[O]	2.83	A:ARG 159[NH2]
59	B:CYS 281[SG]	3.58	A:ILE 121[N]

Salt bridges XML

##	Structure 1	Dist. [Å]	Structure 2
1	B:HIS 75[NE2]	3.94	A:GLU 7[OE1]
2	B:ARG 237[NE]	2.63	A:ASP 86[OD2]
3	B:LYS 149[NZ]	2.71	A:ASP 109[OD1]
4	B:LYS 149[NZ]	3.68	A:ASP 109[OD2]
5	B:HIS 251[NE2]	3.83	A:ASP 111[OD2]
6	B:ARG 218[NH1]	3.62	A:ASP 147[OD1]
7	B:ARG 218[NH1]	3.92	A:ASP 147[OD2]
8	B:ARG 277[NE]	3.60	A:ASP 164[OD2]
9	B:GLU 205[OE1]	3.76	A:ARG 154[NH1]
10	B:ASP 216[OD1]	3.28	A:ARG 107[NH1]
11	B:ASP 216[OD2]	3.40	A:ARG 107[NE]
12	B:ASP 216[OD2]	3.72	A:ARG 107[NH2]

No disulfide bonds found

No covalent bonds found

Supplementary Table S1. Hydrogen bonds and salt-bridges between BMP-1cat and Szl (7EL5 studied herein) in the model complex. BMP1cat corresponds to “structure 2” and Szl to “structure 1” and values have been calculated and listed by the PISA server (PDBePISA v1.52 server).

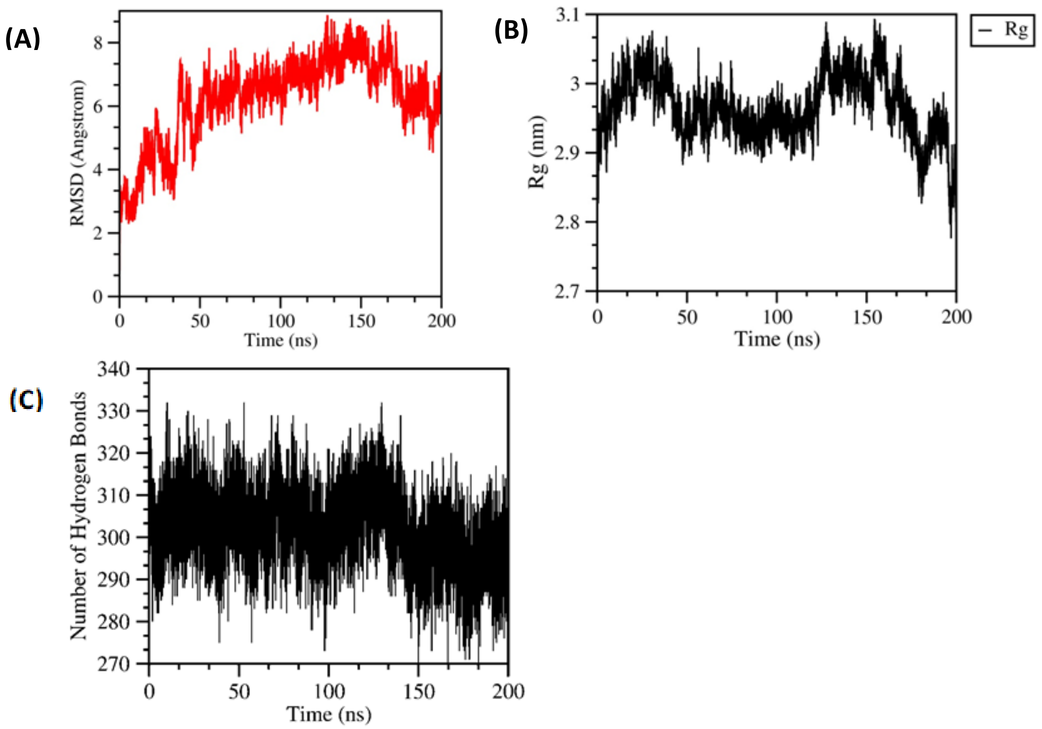

Supplementary Figure S6. Analysis of MD Simulation Parameters of the BMP-1cat/Szl complex during the time course of a 200 ns MD simulation run. (A) Analysis of root mean square deviation (RMSD), (B) Radius of Gyration (C) Average number of hydrogen bonds formed within the BMP-1cat/Szl model complex throughout the simulation run.

Supplementary Table S2. Binding free energy of the modelled BMP1-Szl complex calculated by the Prodigy server using the trajectories from MD simulation runs of 0-200 ns with intervals of 10 ns. The binding free energy, ΔG (kcal/mol), and the dissociation constant, K_D (M), are summarized.

BMP1cat/Szl model complex		
Time (ns)	ΔG_{noelec} (kcal/mol)	Dissociation Constant, K_D (M)
0	-11.7	2.8 E-09
10	-9.8	6.6 E -07
20	-10.8	1.1 E -08
30	-10.2	3.6 E-08
40	-10.1	3.6 E-08
50	-9.9	5.5 E-08
60	-10.2	3.3 E-08
70	-10.7	1.3 E-08
80	-10.5	2.1 E-08
90	-9.8	6.2 E-08
100	-9.8	6.1 E-08
110	-10.7	1.5 E-08
120	-11.6	3.0 E-09
130	-9.4	1.3 E -07
140	-8.3	7.9 E-07
150	-8.6	8.6 E-07
160	-8	1.3 E-06
170	-8.4	6.7 E-07
180	-8.5	5.8 E-07
190	-8.2	9.0 E07
200	-8.7	4.5 E -07
Total	-10.37	0.00
s.d.	1.11	ND

A

B

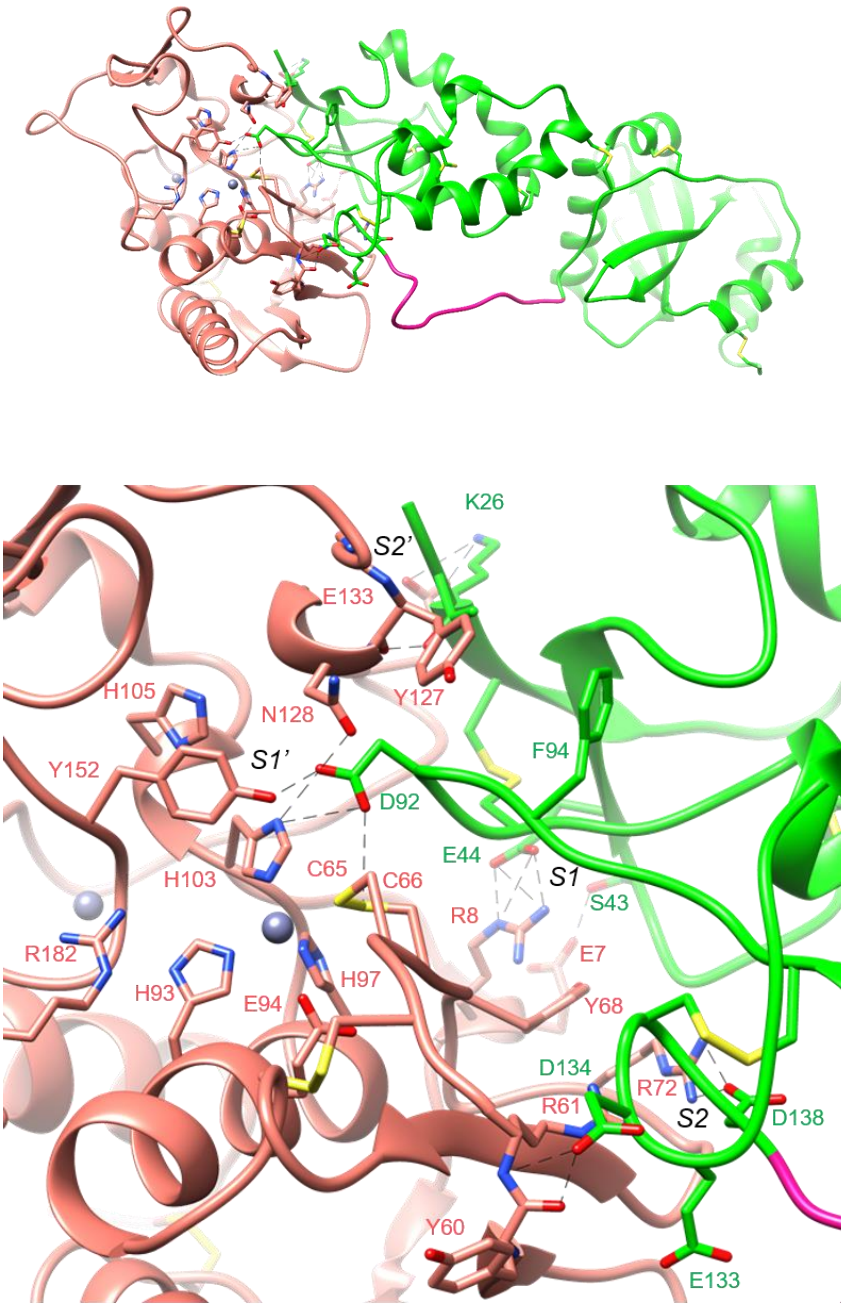

Supplementary Figure S7. Structural model of the BMP-1cat/Szl complex obtained by protein-protein docking. BMP-1cat proteinase is rendered in deep salmon with Szl (in green) docked into the active site cleft. Zinc atoms are indicated by steel blue spheres. (A) Overall view showing the inhibitory loop of Szl fitting deep within the active site cleft of BMP-1. (B) Enlarged view showing amino acids involved in ionic and H-bonded interactions (shown in grey lines) between BMP-1cat and the Szl CRD domain. Interaction subsites are indicated by S2', S1', S1 and S2.

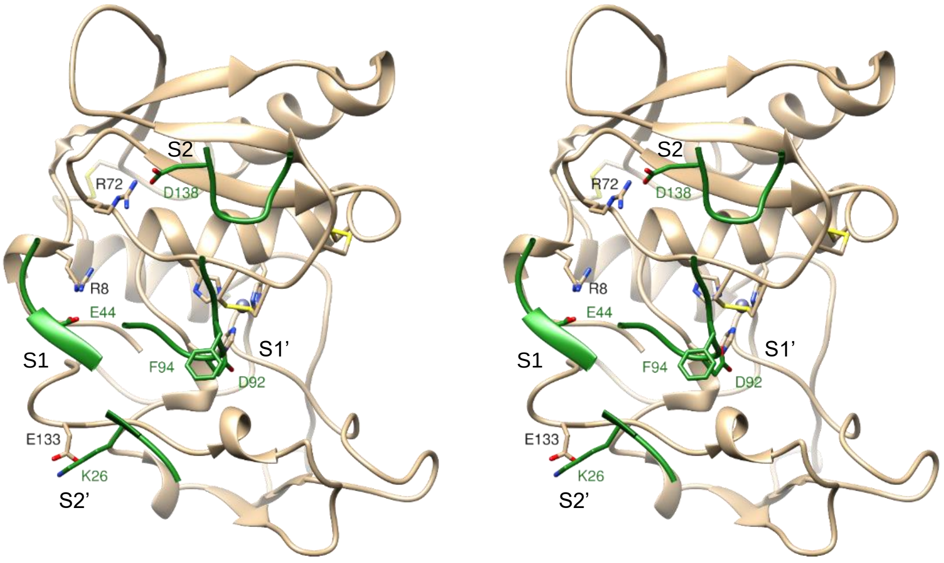

Supplementary Figure S8. Stereo view of BMP-1cat (tan colour) in standard orientation (Guevara et al., 2010) showing (from left to right) the active site cleft running horizontally with histidines bound to the catalytic zinc covered by the vicinal S-S bond. Regions in Szl interacting with BMP-1cat are shown in green with key residues and subsites (S2, S1, S1' and S2') indicated in green and black, respectively.

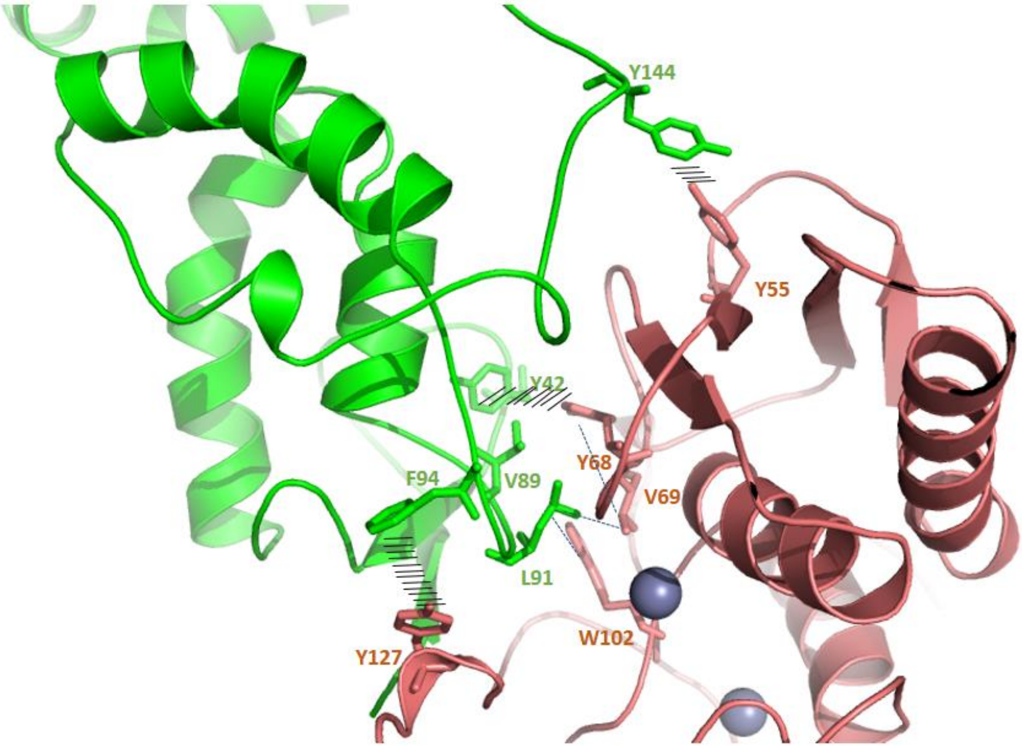

Supplementary Figure S9. Close-up on π-π interactions (wide stippled lines) and hydrophobic interaction (point stippled lines) at the interface of the structural model of the BMP-1cat/Szl complex obtained by protein-protein docking. BMP-1cat proteinase (in deep salmon) with Szl (in green) docked into the active site cleft. Zinc atoms are indicated by steel blue spheres.

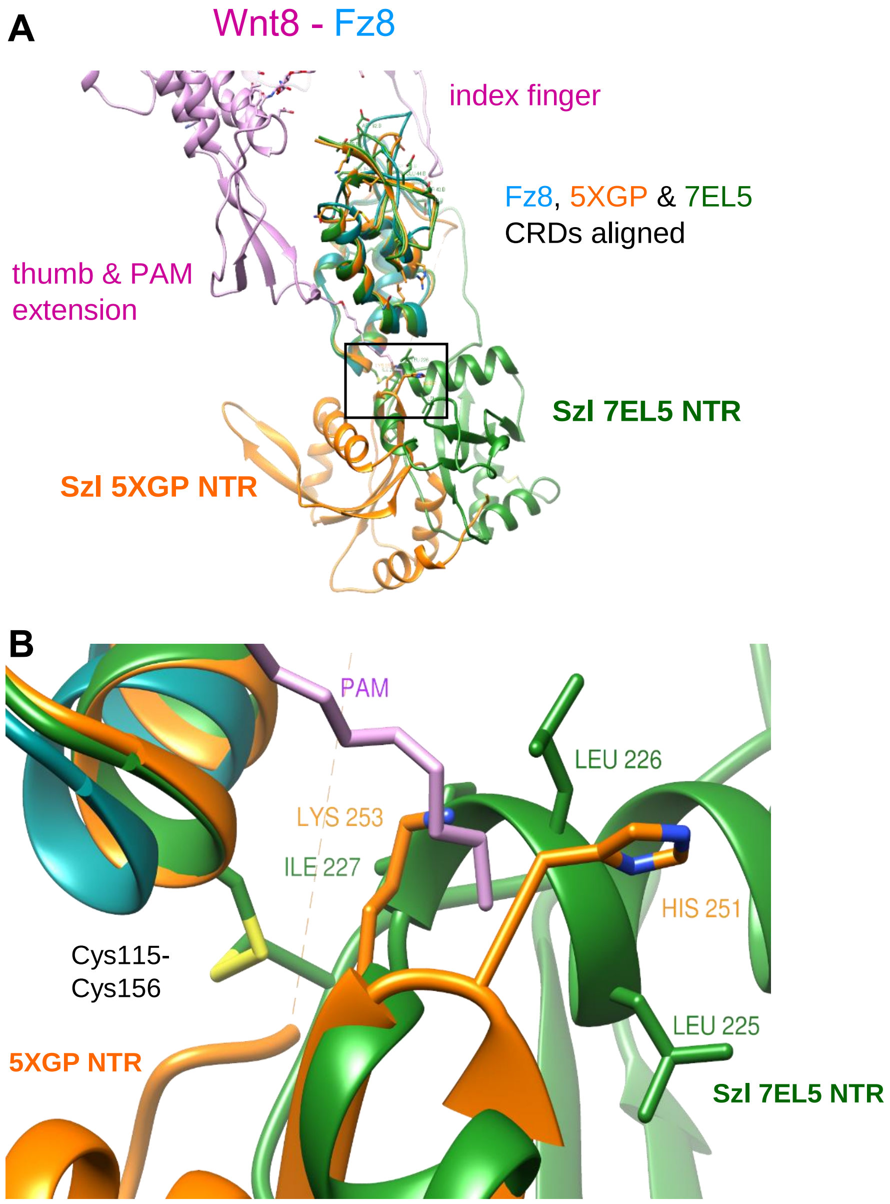

Supplementary Figure S10. Potential interactions of Szl with Wnt8. (A) Overlay of Szl 5XGP (orange, Bu et al., 2017) and Szl 7EL5 (dark green, this work), onto the crystal structure of the Wnt8 (plum) - Fz8 (blue) complex (PDB-ID 4F0A, Janda et al, 2012). Both Szl crystal structures are aligned with Fz8 *via* their CRD domains. (B) Zoom of the region highlighted in (A) showing possible interactions of the hydrophobic PAM (palmitoleic acid) group of Wnt8 with Szl NTR domains. Because of hinge bending, interactions of Szl NTR with PAM are likely to be more favourable for 7EL5.

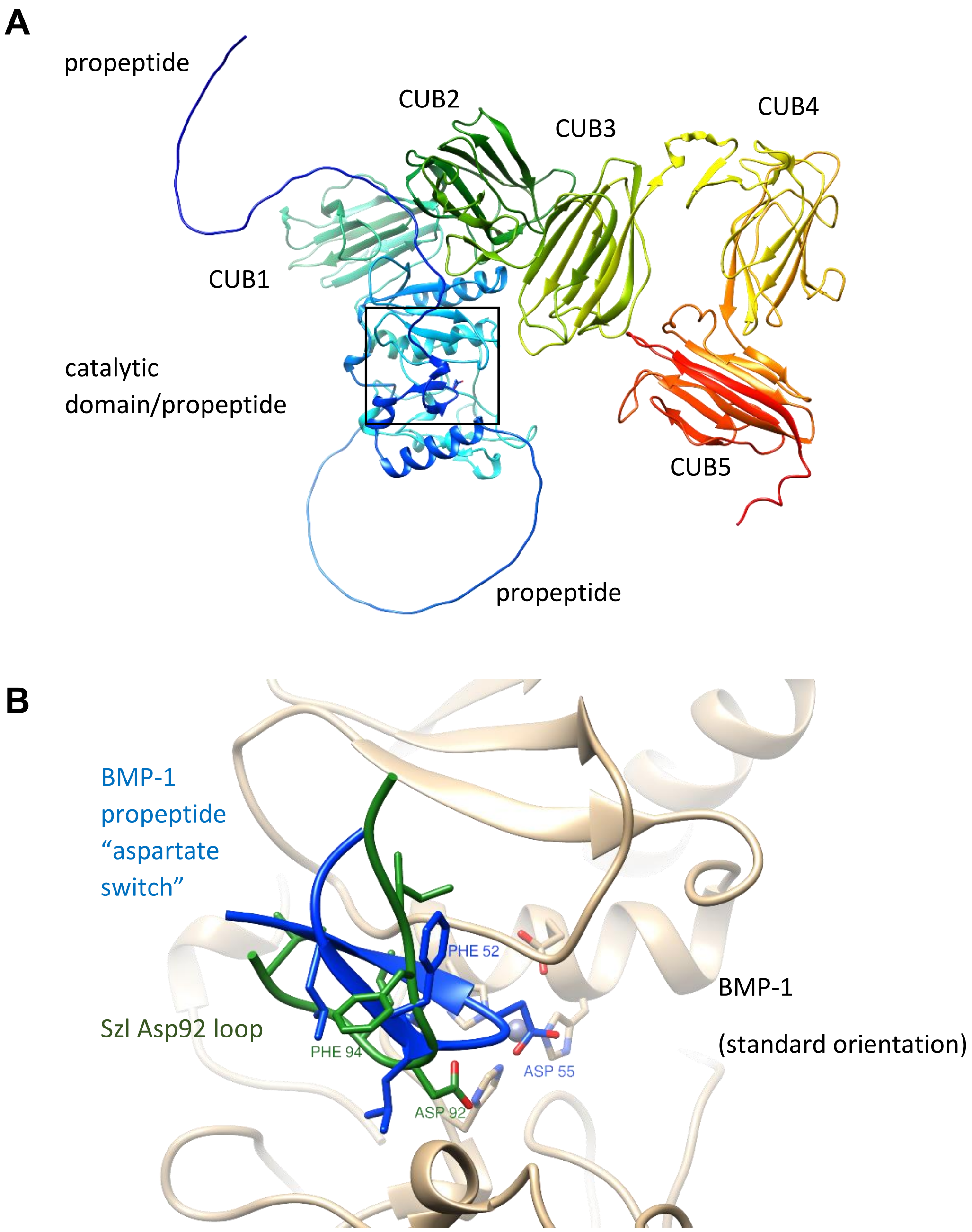

Supplementary Figure S11. Potential interactions of the BMP-1 catalytic domain with the “aspartate switch” in the BMP-1 propeptide region and the Asp92 loop in Szl. (A) Model structure of full-length BMP-1 according to Alpha-Fold2 (AF2) shown using a rainbow colour code from N-terminal blue to C-terminal red. While most of the BMP-1 propeptide is unstructured, AF2 finds the loop containing the “aspartate switch” buried in the BMP-1 catalytic domain (outlined in black). (B) Aspartate switch loop as seen in the region outlined in (A) when the full-length BMP-1 model is superimposed on the BMP-1/Szl model complex from molecular modelling (this study). Structures were aligned using the known structure (PDB ID 3EDH) of the common BMP-1 catalytic domain. The aspartate switch loop in AF2 proBMP-1 (blue) coincides with the Szl Asp92 loop (dark green) found by molecular modelling.

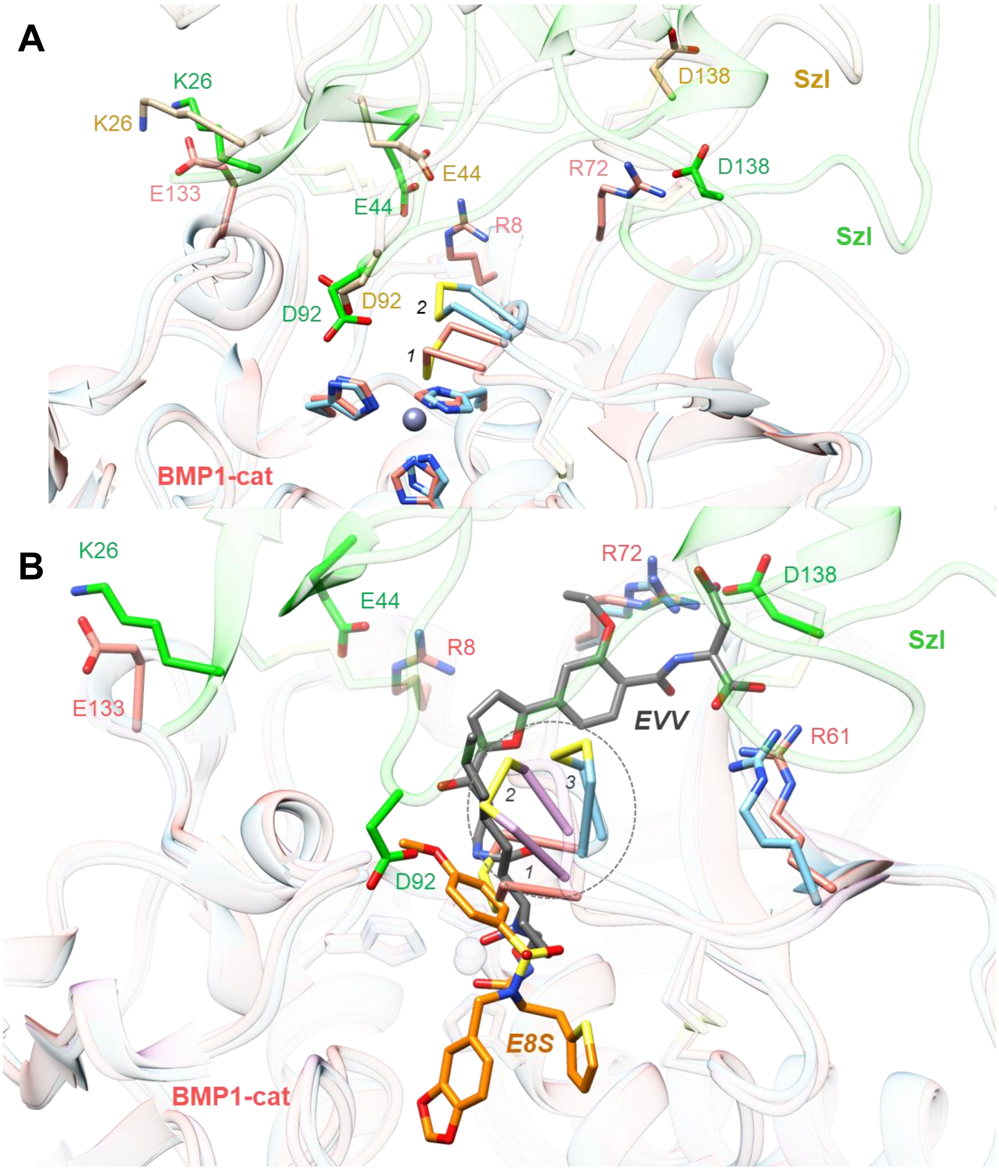

Supplementary Figure S12. Structural model of Szl/BMP-1cat and experimental structures of small molecule inhibitors in complex with the BMP-1 catalytic domain. All structures are aligned using BMP-1cat. (A) Comparison of models of the BMP-1cat/Szl complex with different orientations of the BMP-1cat vicinal S-S bridge (Cys65-Cys66), marked 1 and 2, before and after energy minimization[43], respectively. BMP-1cat and Szl are lightly coloured in salmon pink and green, respectively, in model 1, or plum and tan, in model 2, with key interacting residues in bold. These are close together on the left but further apart on the right, showing how the change in orientation of the disulphide bridge in BMP-1cat leads to a change in the orientation of Szl. Also in bold are the catalytic zinc (grey) and coordinating histidines. (B) Superposition of the BMP-1cat (pale salmon pink) /Szl (green) model reported here with crystal structures (6BTQ, 6BSL) of BMP-1 (plum or blue, respectively) in complex with hydroxamate (E8S, orange) or reverse hydroxamate (EVV, dark grey) inhibitors, with key amino acids and inhibitors in bold. EVV is mostly on the non-prime side of the substrate binding pocket and E8S mostly on the prime side near the catalytic Zn (see (A)). Changes in orientation of the vicinal S-S bridge (Cys65-Cys66) are indicated by the dotted line with numbers 1, 2 and 3 referring to BMP-1cat/Szl, BMP-1cat/6BTQ and BMP-1cat/6BSL, respectively.
